# Unilateral cardiogenic pulmonary edema caused by acute mitral valve prolapse: A case report

**DOI:** 10.1097/MD.0000000000024622

**Published:** 2021-02-19

**Authors:** Xuandong Jiang, Xuping Cheng, Weimin Zhang

**Affiliations:** Intensive Care Unit, Dongyang People's Hospital, Dongyang, Zhejiang, PR China.

**Keywords:** mitral regurgitation, mitral valve prolapse, pneumonia, unilateral pulmonary edema

## Abstract

**Rationale:**

Unilateral cardiogenic pulmonary edema is a rare disease. A common cause is mitral valve and asymmetrical blood regurgitation that is primarily directed toward the upper right pulmonary vein, causing mean capillary pressure to increase on the right side and leading to right pulmonary edema.

**Patient concerns:**

A 41-year-old man was diagnosed with pneumonia after presenting with a 2-day history of cough and shortness of breath. Computed tomography indicated right pulmonary edema. He was managed with noninvasive ventilation; however, his condition continued to deteriorate, and he was transferred to the intensive care unit after tracheal intubation.

**Diagnosis:**

Acute posterior mitral valve prolapses; unilateral cardiogenic pulmonary edema.

**Intervention:**

Emergency mitral valve replacement was performed. During the operation, 2 ruptures of the chordae tendineae in the P2 scallop of the posterior mitral valve were found, and a No. 29 St. Jude mechanical mitral valve was implanted.

**Outcomes:**

Cardiotonic and diuretic drugs were administered postoperatively. Tracheal intubation was removed on day 7; the patient was transferred to the general ward on day 11 and discharged on day 23 postoperatively.

**Lessons:**

Unilateral cardiogenic pulmonary edema is easily misdiagnosed. Computed tomographic (CT) imaging presentation, brain natriuretic peptide, and cardiac color Doppler ultrasound can assist in determining a differential diagnosis. Early surgical treatment is recommended for patients with acute mitral valve prolapse.

## 1 Introduction

Cardiogenic pulmonary edema (CPE) is primarily caused by left ventricular insufficiency and increased hydrostatic pressure.^[1]^ Typical imaging presentation includes symmetrical exudation in the central region of the lungs and a characteristic butterfly shadow. Acute CPE is a very common presentation at our clinic, especially among emergency and critical cases, and it is a serious disease with a high mortality rate that generally presents as bilateral pulmonary edema. Unilateral cardiogenic pulmonary edema (UPE) is rare and easily mistaken on imaging for unilateral lung infiltration diseases such as pneumonia. Study^[2]^ have shown that UPE accounts for about 2% of pulmonary edema cases. UPE is an independent risk factor for patient death, and delayed treatment of this condition may be a reason for its associated increased mortality. We report a case of UPE caused by acute posterior mitral valve leaflet prolapse.

## 2 Case report

**Figure 1:**
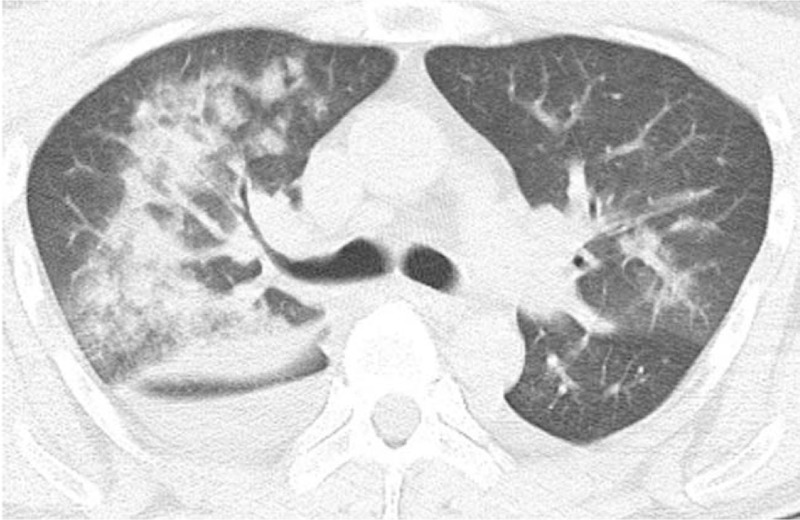
Computed tomographic image when the patient initially reported to our hospital. Exudation in both lungs, which is more significant on the right side, and a small amount of bilateral pleural effusion is observed.

A 41-year-old man was admitted to our hospital with chief complaints of cough and shortness of breath for the past 2 days. He was in good health and had no history of cardiovascular disease. In the emergency room, the patient was conscious, with a temperature of 37.3°, a pulse of 114 beats/minutes, a respiratory rate of 26 breaths/minutes, blood pressure of 110/55 mm Hg, and an SpO_2_ of 83%. Tests and investigations revealed the following: Blood gas analysis, pH 7.44; PCO_2_, 34.9 mm Hg; PO_2_, 60.9 mm Hg; Full blood count–white blood cells, 22.96 × 10^9^/L; neutrophil %, 0.938; C-reactive protein, 20.62 mg/L; procalcitonin, 0.156 ng/ml; high-sensitivity troponin T, 0.057 ng/ml; pro-B-type natriuretic peptide, 1204.0 pg/ml. There were no obvious abnormalities in the cardiac structure, cardiac function, or hemodynamics on echocardiography. His ejection fraction was 65%. Electrocardiography indicated sinus tachycardia (111 beats/minutes). Computed tomography indicated exudation in both lungs, more significant on the right side, and a small amount of bilateral pleural effusion (Fig. 1). Clinical examination revealed bilateral wet rales, regular heart rhythm, and no edema in the lower limbs.

Diagnoses considered were as follows:

1. Severe community-acquired pneumonia and
2. Type I respiratory failure.

The patient was admitted to the respiratory diseases department, and he was managed with noninvasive mechanical ventilation and oseltamivir phosphate antiviral and moxifloxacin antiinfective treatment. However, the patient still had chest tightness and shortness of breath while on the noninvasive ventilator, and his oxygenation level continued to decrease. After an emergency tracheal intubation, the patient was transferred to the intensive care unit. Viral pneumonia was considered, and treatment with human immunoglobulin, ganciclovir, and diuretics was added. During this period, bronchoalveolar lavage, genetic testing, tuberculosis testing, bacterial culture of bronchoalveolar lavage fluid, and various viral tests (syncytial virus, adenovirus, mycoplasma, chlamydia, etc.) did not reveal any significantly abnormal results. The results of pulse index continuous cardiac output monitoring and hemodynamic calculations indicated a cardiac index (CI) of 2.79 L/minutes/m^2^, intrathoracic blood volume index (ITBVI) of 769 ml/minutes/m^2^, global end-diastolic volume index of 616 ml/m^2^, and an extravascular lung water index (EVLWI) of 16.6 ml/kg. However, the patient's condition deteriorated rapidly, and the pulmonary edema worsened. On the third day, EVLWI was 22 ml/kg, and pure oxygen and high positive end-expiratory pressure (PEEP, 15 cm H_2_O) were required to maintain oxygenation; PaO_2_/FiO_2_ dropped to 71 mm Hg. Color Doppler ultrasound reexamination indicated posterior mitral valve leaflet prolapse, possible rupture of the chordae tendineae of the posterior mitral valve, and moderate to severe mitral regurgitation (MR), Mild tricuspid regurgitation and Mild enlargement of left atrium, EF: 65%. Emergency mitral valve replacement was performed, and 2 ruptures of the chordae tendineae in the P2 scallop of the posterior mitral valve were found. The anterior valve was elongated and poorly aligned, the annulus was significantly enlarged, and regurgitation was incomplete; mitral valve replacement was confirmed. The root of the anterior mitral valve leaflet was opened, the leaflets of the mitral valve were preserved, and a No. 29 St. Jude mechanical mitral valve was implanted.

**Figure 2:**
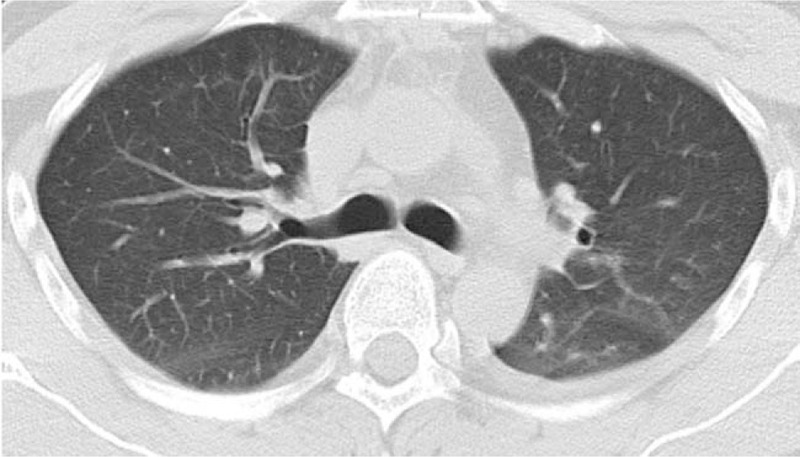
Computed tomographic image on postoperative day 18 (before the patient was discharged). The pulmonary edema is almost resolved.

Cardiotonic and diuretic drugs were administered postoperatively. Tracheal intubation was removed on day 7. The patient was transferred to the general ward on day 11 and discharged on day 23 postoperatively. A repeated computed tomography, taken before patient was discharged, showed an almost complete resolution of the pulmonary edema (Fig. 2). Thereafter, outpatient reexamination at our hospital over the next 3 months indicated a good cardiopulmonary function.

## 3 Discussion

The main cause of UPE is severe MR. Most cases of UPE occur primarily on the right side, as seen in our patients and in another report.^[3]^ Its pathophysiology may be asymmetrical blood regurgitation in MR, especially in patients with mitral valve prolapse, caused by acute chordal rupture. The regurgitated blood is primarily directed toward the upper right pulmonary vein, causing the mean capillary pressure to increase on the right side and leading to right pulmonary edema.^[4]^ Another explanation is that regurgitation causes a sudden increase in left atrial pressure, after which the lymphatic drainage capacity of the right lung becomes worse than that of the left, which can lead to edema and induce right UPE.^[5]^

UPE is easily misdiagnosed, resulting in delayed treatment. When our patient was admitted to the hospital, we heard bilateral wet rales, which greatly obscured the systolic murmur in the mitral valve auscultation area. Therefore, ultrasound examination is essential. However, emergency color Doppler ultrasound of our case did not indicate MR, and the possibility of a poor image quality from the emergency department bedside ultrasound was considered. On day 3, we consulted with a senior ultrasonography radiologist who found a posterior mitral valve leaflet prolapse and possible rupture of the chordae tendineae of the posterior mitral valve. If bedside ultrasound B-scan cannot confirm a diagnosis of MR, there have been reports^[6,7]^ that the use of transesophageal echocardiography can be used to assess the degree of MR more accurately and reduce misdiagnosis.

CPE is characterized by a sharp increase in left ventricular filling pressure, which increases the amount of fluid leaking into the lung interstitium and alveolar cavity, thereby causing pulmonary edema. We performed pulse index continuous cardiac output monitoring of our patient, with which EVLWI was used to quantify the severity of pulmonary edema. Even with the addition of a proactive diuretic drug treatment, EVLWI increased from 16.6 to 22 ml/kg and continued to increase thereafter. Monitoring EVLWI has significance for guiding clinical treatment. In addition, the elevated probrain natriuretic peptide (BNP) level in our patient also suggested the possibility of CPE. At that time, we considered myocardial damage caused by severe viral infection leading to the increase of BNP levels. However, a prospective study^[8]^ showed that both BNP and proBNP could be used to distinguish CPE from nonCPE and that BNP has a superior diagnostic effect. Therefore, BNP is required for monitoring patients with pulmonary edema.

CT has a better imaging resolution than chest X-ray. CPE can be distinguished from non-CPE on imaging,^[9]^ and it is characterized by the following:

1. Redistribution of lung texture; increased texture and blurring of the upper lung and outer band of the lung;
2. Thickened bronchovascular bundles (peribronchial vascular edema);
3. Thickened interlobular septa;
4. The center is more obvious than the periphery, namely a butterfly shadow or bat-wing opacities;
5. Basic manifestations of heart disease, such as cardiomegaly and coronary artery calcification.

For patients with acute MR, an immediate surgical intervention is recommended, but the risks of acute MR surgery are very high. A multi-center study involving 279 patients who underwent emergency surgery for acute severe MR reported a mortality rate of 22.5%.^[10]^ Therefore, the primary goal of the medical treatment of acute MR is to stabilize the patient's condition and prepare the patient for surgery. Sodium nitroprusside can reduce systemic vascular resistance, thereby alleviating MR; however, the use of vasodilators in patients with systemic hypotension is usually limited. Moreover, the short-term circulatory assistive devices,^[11]^ such as the intra-aortic balloon counterpulsation, Impella percutaneous left ventricular assist device, and extracorporeal membrane oxygenation (ECMO), can be used as a temporary measure before emergency valve surgery to temporarily stabilize the patient's condition. After 3 days of medical treatment, our patient's condition had not improved. His oxygenation level fell to 71 mm Hg, which was an indication for ECMO treatment; however, as we prepared for ECMO treatment, the patient's condition fortunately improved and ECMO treatment was not necessary. This consequently avoided a series of possible complications associated with ECMO. Our patient had an obvious rupture of the chordae tendineae of the posterior mitral valve during the operation. 2017 ESC/EACTS guidelines recommend immediate surgical treatment for MR caused by symptomatic acute severe chordal rupture.^[12]^ Valve repair may be selected as the surgical method. Compared with valve replacement, mitral valve repair has a lower perioperative mortality, preserves left ventricular function, and results in better long-term survival. In addition, long-term oral anticoagulation drug therapy may be avoided after artificial valve replacement. The possibility of valve repair was evaluated during the operation, and ultimately, mitral valve replacement was selected.

## 4 Conclusion

UPE is easily misdiagnosed, and timely diagnosis is essential. CT imaging findings, BNP, and cardiac color Doppler ultrasound may be used for differential diagnosis. Mitral valve regurgitation is the most common cause. Surgery is recommended; however, the intraoperative and postoperative risks of emergency surgery are extremely high. ECMO may be used to support transitional treatment.

## Acknowledgments

We would like to acknowledge Dr Yunxiao Lv for his invaluable assistance. In addition, we would like to thank Editage (www.editage.cn) for English language editing.

## Author contributions

**Conceptualization:** Xuandong Jiang.

**Formal analysis:** Xuandong Jiang.

**Validation:** Weimin Zhang.

**Writing – original draft:** Xuandong Jiang.

**Writing – review & editing:** Xuandong Jiang, Xuping Cheng, Weimin Zhang.
